# On the importance of crystal structures for organic thin film transistors

**DOI:** 10.1107/S2053229624008283

**Published:** 2024-09-04

**Authors:** Guillaume Schweicher, Susobhan Das, Roland Resel, Yves Geerts

**Affiliations:** a Université Libre de Bruxelles (ULB) Faculté des Sciences Laboratoire de chimie des polyméres Boulevard du Triomphe 1050 Bruxelles Belgium; b Institute of Solid State Physics, Graz University of Technology, Petersgasse 16, 8010 Graz, Austria; c Université Libre de Bruxelles (ULB), International Solvay Institutes of Physics and Chemistry, Boulevard du Triomphe, 1050 Bruxelles, Belgium; d WEL Research Institute, avenue Pasteur 6, 1300 Wavre, Belgium; University of Strathclyde, United Kingdom

**Keywords:** organic semi­con­duc­tor, electronic properties, organic thin film transistors, charge carrier mobility, crystal structure

## Abstract

Historically, knowledge of the mol­ecular packing within the crystal structures of organic semi­con­duc­tors has been instrumental in understanding their solid-state electronic properties. Nowadays, crystal structures are thus becoming increasingly important for enabling engineering properties, understanding poly­mor­phism in bulk and in thin films, exploring dynamics and elucidating phase-transition mech­a­nisms. This review article introduces the most salient and recent results of the field.

## Abstract 2

Knowledge of the mol­ecular packing within the crystal structures of organic semi­con­duc­tors has been instrumental in understanding their solid-state electronic properties. Crystal structures are thus becoming increasingly important for enabling engineering properties, understanding poly­mor­phism in bulk and in thin films, exploring dynamics and elucidating phase-transition mech­a­nisms.

## Introduction

**Figure 1:**
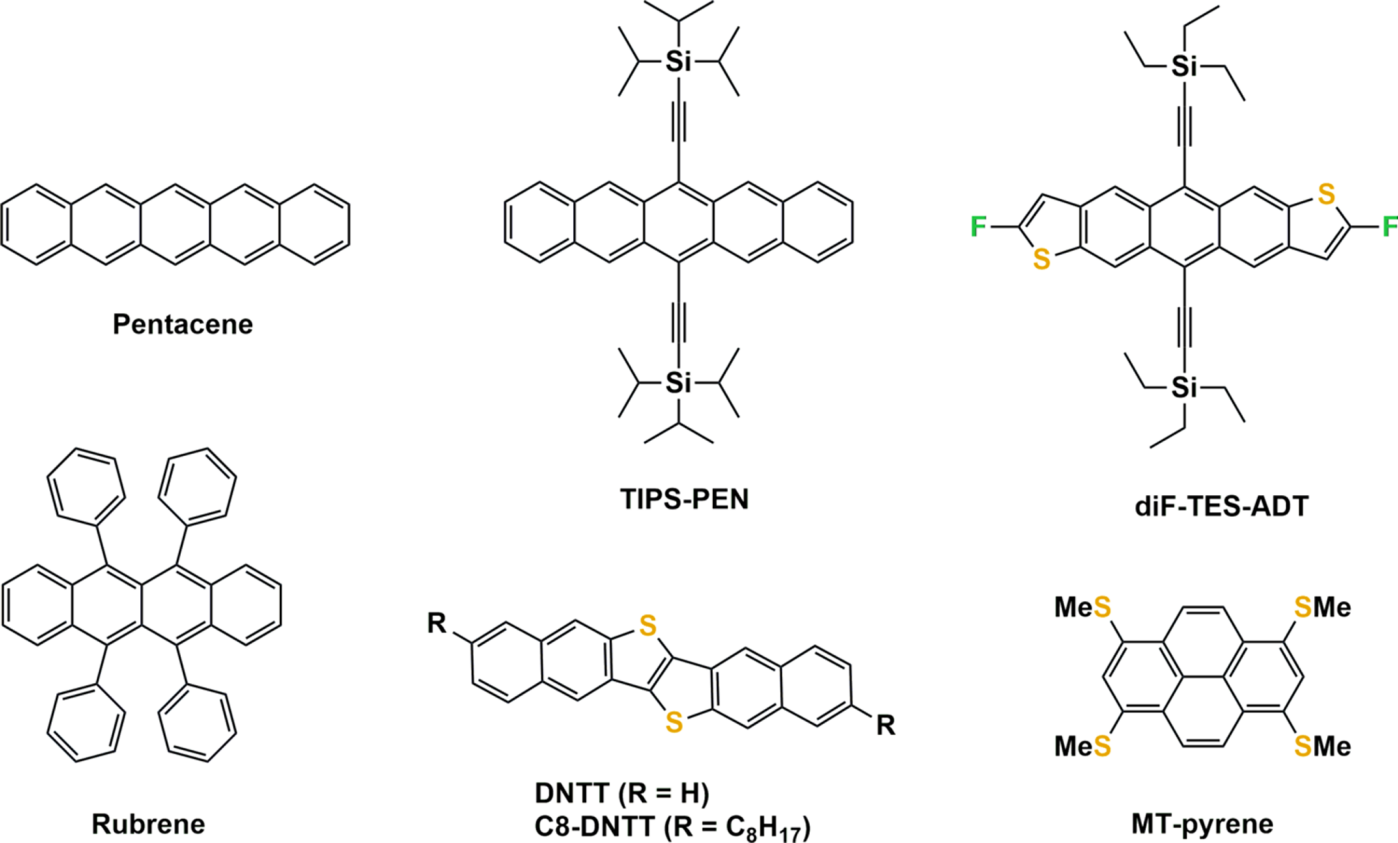
A selection of some good-performing and historically important mol­ecular semi­con­duc­tors: penta­cene (Mas-Torrent & Rovira, 2011 ▸), rubrene (Menard *et al.*, 2004 ▸; Sundar *et al.*, 2004 ▸), 6,13-bis(triiso­propyl­silylethyn­yl)penta­cene (TIPS-PEN) (Anthony *et al.*, 2001 ▸), 2,8-di­fluoro-5,11-bis­(tri­ethyl­silylethyn­yl)anthradi­thio­phene (diF-TES-ADT) (Subramanian *et al.*, 2008 ▸), di­naphtho­[2,3-*b*:2′,3′-*f*]thieno[3,2-*b*]thio­phene (DNTT) (Takimiya *et al.*, 2014 ▸), dioctyldi­naphtho­[2,3-*b*:2′,3′-*f*]thieno[3,2-*b*]thio­phene (C8-DNTT) (Kang *et al.*, 2011 ▸) and 1,3,6,8-tetra­kis­(methyl­thio)­pyrene (MT-pyrene) (Takimiya *et al.*, 2021 ▸).

The field of organic electronics relies on the use of organic com­pounds as semiconducting materials for the transport of either electrons (e^−^) or holes (h^+^) to perform logic operations and to convert light into electrical current or *vice versa*. Organic semi­con­duc­tors are π-conjugated systems that are often flat and rigid. Two classes of organic semi­con­duc­tors exist: polymers and low-mol­ecular-weight com­pounds (Ostroverkhova, 2016 ▸). Macromolecules provide a fast lane for charge transport along their π-conjugated backbone, but charge hopping between chains is much slower which limits charge carrier mobility μ (cm^2^ V^−1^ s^−1^). The latter is defined as the drift velocity of the charge carrier (cm s^−1^) per unit applied electric field (V cm^−1^) and serves as the figure of merit for benchmarking semi­con­duc­tor performance. π-Conjugated polymers are only semicrystalline, meaning that some ordered and amorphous regions co-exist within the same sample, but also within the same macromolecular chain (Ding *et al.*, 2023 ▸). Crystalline order is essential for efficient charge transport; for example, single-crystal silicon (μ = 1200 cm^2^ V^−1^ s^−1^) exhibits a charge carrier mobility three times greater than amorphous films (μ = 1 cm^2^ V^−1^ s^−1^) (Schweicher *et al.*, 2020 ▸). Low-mol­ecular-weight organic semi­con­duc­tors generally show higher charge carrier mobility values than π-conjugated polymers, which is a result of their crystallinity (Sawatzki-Park *et al.*, 2023 ▸). The fact that mol­ecular semi­con­duc­tors can com­pletely crystallize paves the way to com­pre­hen­sive studies on the role of mol­ecular structure, crystal packing and crystal–lattice dynamics in charge transport (Fratini *et al.*, 2020 ▸; Giannini & Blumberger, 2022 ▸). Notably, charge-transport studies on single crystals are particularly instructive for es­tab­lishing reliable structure–property relationships (Ger­shen­son *et al.*, 2006 ▸; Liu *et al.*, 2011 ▸; Takimiya *et al.*, 2014 ▸; Iino *et al.*, 2015 ▸; Fraboni *et al.*, 2016 ▸; Tsurumi *et al.*, 2017 ▸; Onwubiko *et al.*, 2018 ▸; Wang *et al.*, 2018 ▸; Yamamura *et al.*, 2018 ▸; Zhang *et al.*, 2018 ▸; He *et al.*, 2018 ▸). As can be seen in Fig. 1 ▸, which presents a selection of good-performing organic semi­con­duc­tors, solubilizing side groups are often linked to π-conjugated cores. However, side groups do much more than just confer solubility in common organic solvents. They are also essential for engineering crystal structures that majorly impact the transfer integrals *J* (meV), which qu­antify the extent of wave function overlap between adjacent π-systems (Anthony, 2006 ▸, 2008 ▸; Coropceanu *et al.*, 2007 ▸; Liu *et al.*, 2011 ▸; Mas-Torrent & Rovira, 2011 ▸; Mitsui *et al.*, 2014 ▸; Schweicher *et al.*, 2014 ▸, 2020 ▸; Yu *et al.*, 2019 ▸; Jiang & Hu, 2020 ▸; Okamoto *et al.*, 2020 ▸; Takimiya *et al.*, 2024 ▸). In principle, the higher the value of *J*, the larger the value of μ. However, it is a necessary, but not sole, condition since lattice dynamics and the dimensionality of transport [one- (1D), two- (2D) or three-dimensional (3D)] also play major roles in the performance of semi­con­duc­tors (Sundar *et al.*, 2004 ▸; Schweicher *et al.*, 2015 ▸; Illig *et al.*, 2016 ▸). Static and dynamic disorder force charges to localize which substanti­ally decreases charge carrier mobility (Fratini *et al.*, 2016 ▸; Tsutsui *et al.*, 2016 ▸; Schweicher *et al.*, 2019 ▸; Banks *et al.*, 2023 ▸). Finally, it is worth mentioning that thermal expansion coefficients along different crystallographic directions are instrumental for studying and understanding the temperature dependence of charge-transport properties (Li *et al.*, 2012 ▸, 2013 ▸; van der Lee *et al.*, 2018 ▸; Jouclas *et al.*, 2022 ▸). This review focuses on the link between the crystal structures of mol­ecular semi­con­duc­tors and their charge-transport properties. It is not the ambition to be com­pre­hen­sive due to the many types of mol­ecules and crystal structures in this field. However, it highlights the importance of crystallographic information for characterizing and understanding solid-state properties using some representative examples, mostly, but not exclusively, from our research groups (Rivnay *et al.*, 2012 ▸).

## Mol­ecular structure assessment

**Figure 2:**
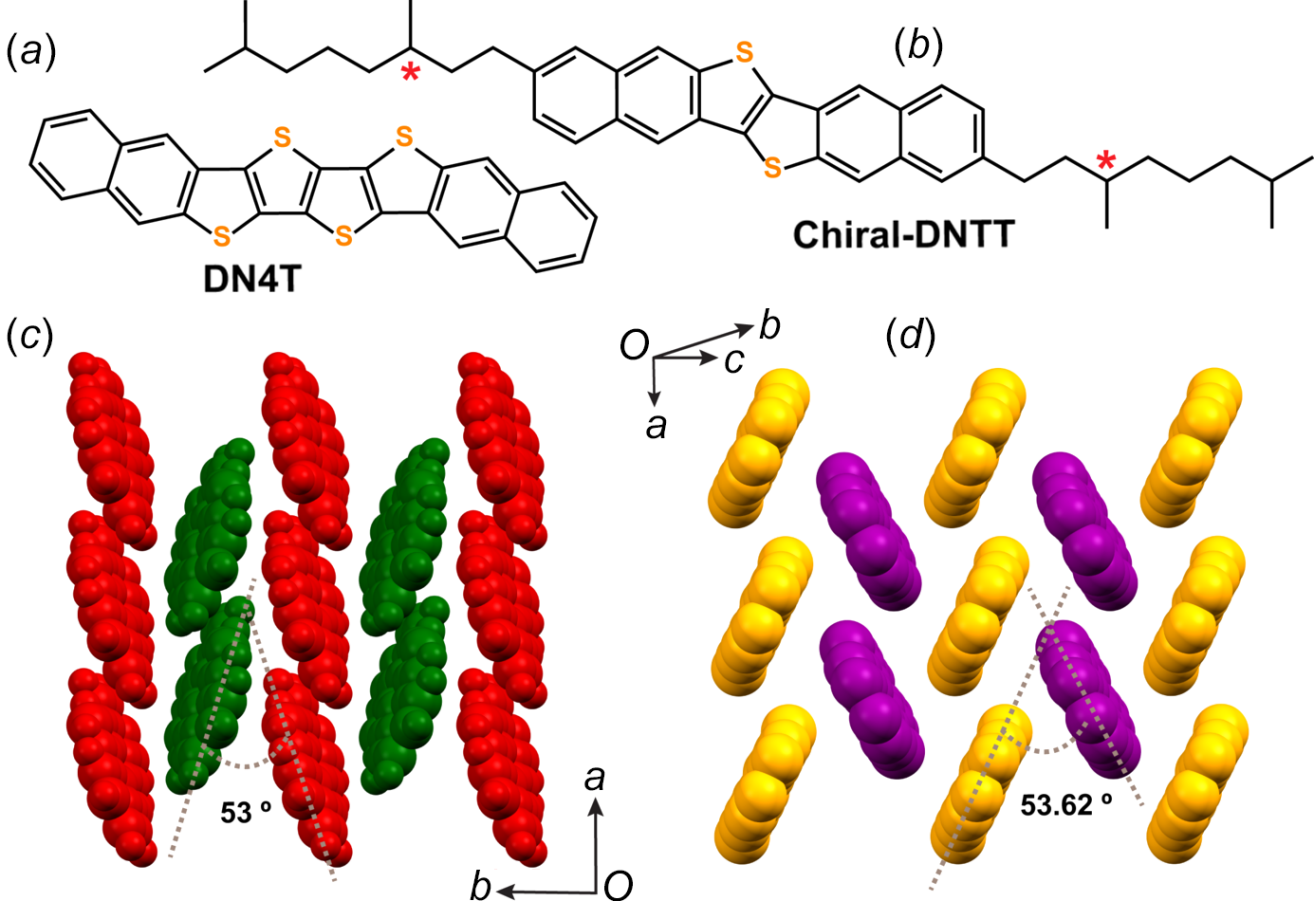
The mol­ecular structure of (*a*) DN4T and (*b*) chiral DNTT. (*c*)/(*d*) The corresponding crystallographic herringbone packing, defining the change in the dihedral angle, ultimately determined by solving the crystal structures. The chiral supra­molecular arrangement of the achiral DNTT core induced by the chiral alkyl side chains is indicated (marked by red asterisks) (Volpi *et al.*, 2023 ▸).

Materials chemists synthesizing organic semi­con­duc­tors rely heavily on proton and carbon nuclear magnetic resonance (^1^H and ^13^C NMR) and on UV–visible (UV–Vis) spectroscopy for assessing mol­ecular structures (Takimiya *et al.*, 2014 ▸; Anthony, 2008 ▸). These methods require, however, a minimum solubility in common organic solvents that nonsubstituted extended π-systems rarely possess. Mass spectrometry, which can be performed on powders, provides valuable information on parent ions and fragmentation fingerprints that give hints to the mol­ecular structures, but which cannot be considered as sufficient proof itself. Despite the fact that nonsubstituted extended π-systems are rather insoluble, their high thermal stability allows their sublimation under high vacuum (Jiang & Kloc, 2013 ▸). In many cases, these π-systems form, from the vapour phase, high-quality crystals that are ideal for crystal structure determination by X-ray diffraction (Zhang *et al.*, 2018 ▸). On the other hand, extended π-systems substituted by alkyl chains or other side groups allow the production of single-crystal domains through conventional or advanced solution-processing methods in the event of decent solubility in conventional solvents (Diao *et al.*, 2014*b* ▸; Yamamura *et al.*, 2018 ▸). Single crystals obtained from solution are equally suitable for structure solution by X-ray diffraction. Poor solubility combined with poor thermal stability will lead to a more challenging crystal structure determination. In some cases, the electron diffraction (ED) analysis of micron-sized or even submicron-sized crystals can help in solving a structure (Altoe *et al.*, 2012 ▸; Gruene *et al.*, 2021 ▸; Brázda *et al.*, 2019 ▸). ED also provides information on crystal disorder and dynamics (Illig *et al.*, 2016 ▸). In general, crystal structures offer the ultimate evidence of mol­ecular identity when solution characterization methods are inoperative, as illustrated for DN4T in Fig. 2 ▸(*a*) (Jouclas *et al.*, 2022 ▸). Importantly, this also holds true for assessing stereochemistry at the mol­ecular and supra­molecular levels. Fig. 2 ▸(*b*) shows the chiral supra­molecular packing of the achiral DNTT core due to the presence of chiral alkyl side chains (Volpi *et al.*, 2023 ▸).

## Inter­molecular and electronic inter­actions

Crystal structures contain a wealth of information that is particularly valuable for assessing the inter­molecular inter­actions between adjacent mol­ecules. The relative positions of π-systems, the inter­molecular distances, the mol­ecular volume and the number of crystallographically different mol­ecules per unit cell originate from the delicate balance between attractive and repulsive inter­molecular inter­actions (Sutton *et al.*, 2016 ▸). Various types of attractive electrostatic inter­molecular inter­actions are at play, such as hydrogen bonding, van der Waals forces and quadrupolar inter­actions. Inter­molecular repulsion, explained by the Pauli exclusion principle, is far from being negligible as it accounts for mol­ecular shape (Israelachvili, 2011 ▸). To deal with the com­plexity and the amount of information on inter­molecular inter­actions within crystal structures, com­puting tools, such as fingerprint plots and Hirshfeld surfaces, are nowadays used routinely (Spackman & McKinnon, 2002 ▸; Spackman & Jayatilaka, 2009 ▸; Spackman, 2013 ▸; Niebel *et al.*, 2015 ▸). Crystal structures are also exploited to calculate the above-mentioned transfer integrals (Coropceanu *et al.*, 2007 ▸; Fratini *et al.*, 2020 ▸). There exists, however, no general correlation between the relative positions and the inter­molecular distances of π-systems with *J* values since the latter are determined by the extent of wave function overlap between adjacent mol­ecules (Fratini *et al.*, 2017 ▸). Transfer integrals assume values in the range 0–100 meV, while significantly larger values of up to 800 meV are encountered in disk-like mol­ecules such as phthalocyanines (Tant *et al.*, 2005 ▸). Transfer integral patterns are orbital specific. Notably, *J* value patterns for highest occupied mol­ecular orbitals (HOMOs) and lowest unoccupied mol­ecular orbitals (LUMOs) differ considerably (Brédas *et al.*, 2002 ▸). Crystal structures also allow the calculation of intra­molecular (local) and inter­molecular (nonlocal) phonon modes that are essential for understanding the lattice dynamics within crystals (Bedoya-Martínez *et al.*, 2017 ▸; Schweicher *et al.*, 2019 ▸; Jouclas *et al.*, 2022 ▸; Banks *et al.*, 2023 ▸). Slow inter­molecular vibrations, corresponding to 10–150 cm^−1^ phonon modes, limit charge transport by inducing a fluctuating structural disorder causing the transfer integrals to adopt a wide distribution of values at room temperature (Fratini *et al.*, 2016 ▸). Consequently, charge carriers get transiently localized over clusters of π-systems, resulting in charge carrier mobility values on the order of 10 cm^2^ V^−1^ s^−1^ over long time and length scales. The situation differs radically at short time and length scales, *i.e.* 10^−10^ s and 100 nm, respectively. Under these conditions, the charge carrier mobility exceeds 100 cm^2^ V^−1^ s^−1^ (Tsutsui *et al.*, 2016 ▸; Giannini *et al.*, 2023 ▸). The resulting localization length is determined by the crystal structure, phonon modes and temperature (Giannini *et al.*, 2023 ▸). Crystal dynamics and, in particular, some specific inter­molecular phonon modes, also trigger crystal-to-crystal phase transitions (*vide infra*) (Chung *et al.*, 2018*a* ▸; Asher *et al.*, 2022 ▸, 2023 ▸; Ferrari *et al.*, 2023 ▸). In any case, crystal structures are the essential inputs for many further studies. Note, however, that crystal structures do not provide all information regarding charge transport. Notably, crystal twisting, *i.e.* the formation of helical ribbons, with repetitive optical textures in the 1–100 µm range, also impacts charge transport (Yang *et al.*, 2022 ▸, 2024 ▸; Whittaker *et al.*, 2023 ▸).

## Crystal structure engineering

**Figure 3:**
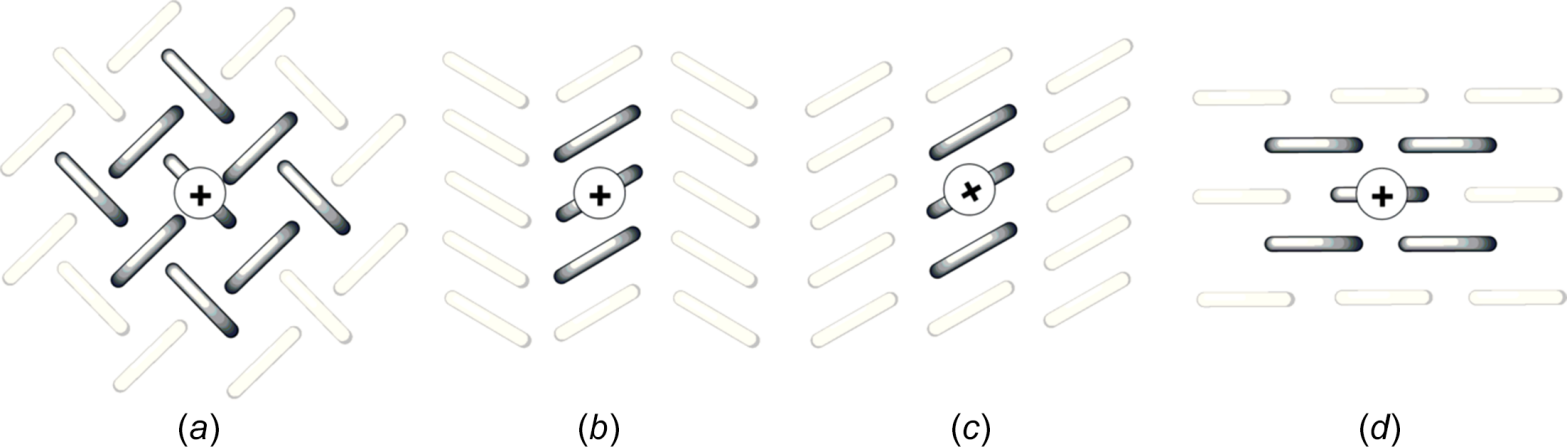
Schematic illustration of the most encountered crystalline packing motifs of mol­ecular semi­con­duc­tors. The mol­ecules bearing a plus (+) sign and their first neighbours are strongly connected electronically and are represented in dark grey. (*a*) The herringbone arrangement with charge transport dominated by edge-to-face inter­actions, (*b*) slipped π-stacking, (*c*) slipped-stack packing and (*d*) a brick-wall arrangement.

**Figure 4:**
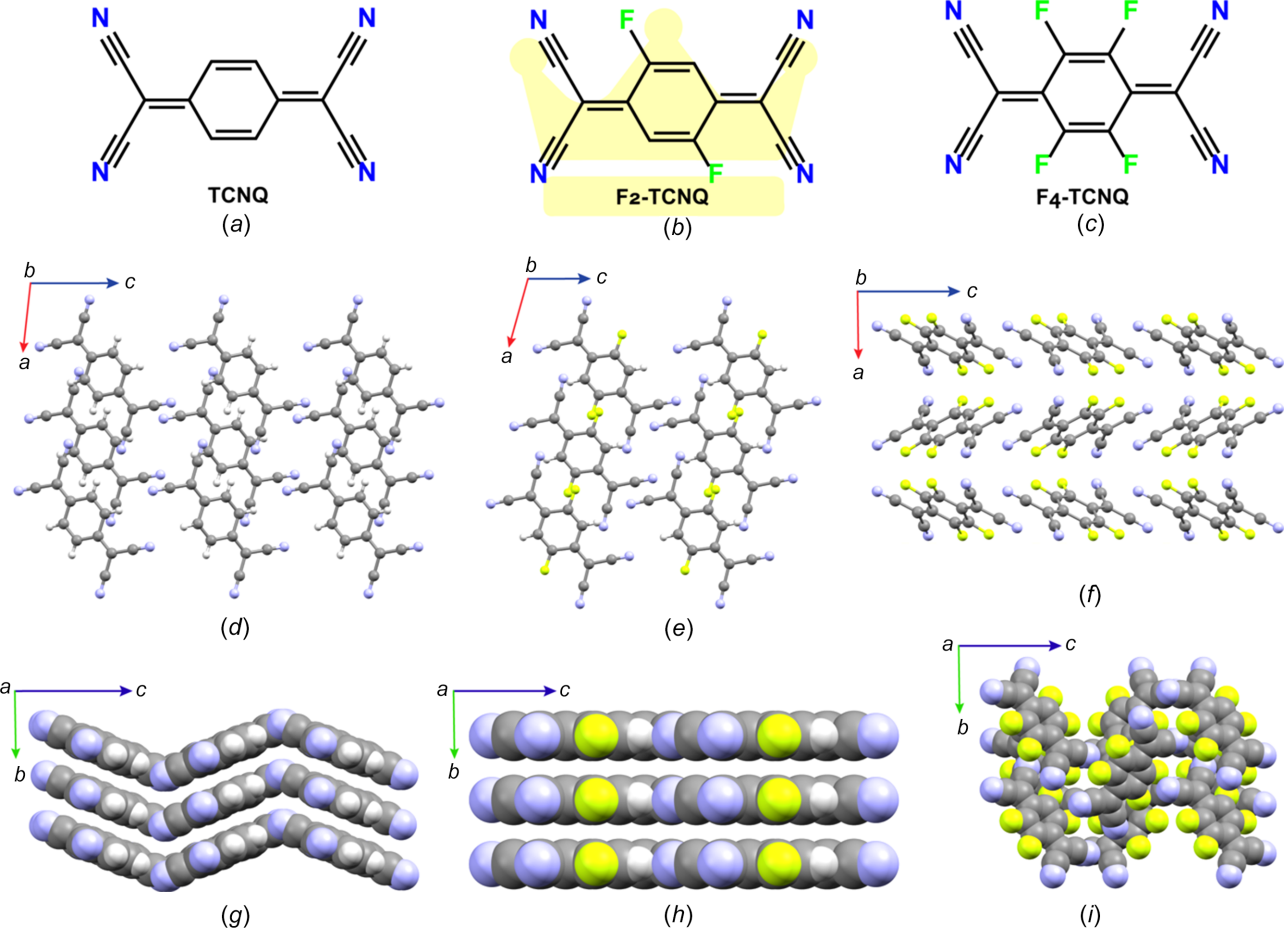
The mol­ecular structures and a com­parison of the crystallographic packing in the TCNQ family for (*a*) TCNQ, (*b*) F2-TCNQ and (*c*) F4-TCNQ. The mol­ecular packing viewed along different crystallographic directions for (*d*)/(*g*) TCNQ, (*e*)/(*h*) F2-TCNQ and (*f*)/(*i*) F4-TCNQ. Note that F2-TCNQ has the superior transporting nature [highlighted by a coloured crown in the background in part (*b*)] within the family.

As the performance of mol­ecular semi­con­duc­tors depends jointly on their mol­ecular identity and packing, chemists have engineered crystal structures that are categorized into four main groups, as shown in Fig. 3 ▸ (no sharp borders separate them). Note that other structural arrangements, such as sandwich herringbone packing, also exist, but they are less often encountered (Desiraju & Gavezzotti, 1989 ▸). The key design concept for crystal structures is to maximize the dimensionality of charge transport. One-dimensional (1D) semi­con­duc­tors, *i.e.* when charge transport is efficient only along one specific crystallographic orientation, perform less well than two-dimensional (2D) semi­con­duc­tors, which are more resilient to defects and allow the delocalization of charge carriers over a larger number of mol­ecules (Skabara *et al.*, 2013 ▸). Another important feature of the performance of organic devices is the thin film quality (Virkar *et al.*, 2010 ▸). In general, brick-wall and herringbone arrangements tend to yield better thin films made of plate-like crystals (Wang *et al.*, 2018 ▸). Materials chemists have mostly played with the size, shape and volume of electronically inert side groups for reaching the desired 2D charge transport inherent in herringbone and brick-wall arrangements, as illustrated in Figs. 3 ▸(*a*) and 3(*d*), respectively (Wang *et al.*, 2018 ▸; Takimiya *et al.*, 2021 ▸). This is probably best illustrated by the TIPS-PEN and diF-TES-ADT cases (see Fig. 1 ▸). Both mol­ecules form p-type semi­con­duc­tors because they preferentially transport h^+^ over e^−^. The steric demand of the inert triiso­propyl­silylethynyl side groups of TIPS-PEN allows the positioning of the penta­cene core in a brick-wall arrangement that is the most favourable for charge transport because h^+^ can move along several pathways. Smaller or larger side groups fail to provide the necessary bulkiness for reaching the desired brick-wall arrangement (Anthony *et al.*, 2001 ▸). The mol­ecular design is even more elaborate for diF-TES-ADT, for which the best packing is reached through the steric demand of the tri­ethyl­silylethynyl side groups, *i.e.* slightly less than for TIPS-PEN, because the antradi­thio­phene core has a lower volume than the penta­cene core, but also *via* attractive inter­actions between the F and S atoms. Such precise mol­ecular engineering work would have simply been impossible without the wealth of information provided by the solution of crystal structures (Subramanian *et al.*, 2008 ▸). The same concept of optimizing crystal packing by side groups also applies to C8-DNTT and MT-Pyrene (Takimiya *et al.*, 2011 ▸, 2021 ▸). A slightly different approach consists in varying the position and number of substituents on π-conjugated cores, as illustrated in Fig. 4 ▸ for TCNQ, F2-TCNQ and F4-TCNQ. For these com­pounds also, the crystal structures have been essential in understanding why F2-TCNQ stands out as an exceptional n-type semi­con­duc­tor, preferentially transporting e^−^ over h^+^, where­­as TCNQ and F4-TCNQ perform rather poorly (Krup­skaya *et al.*, 2015 ▸; Shukla *et al.*, 2019 ▸). The F2-TCNQ case shows that crystal structure elucidation enables much more than simply a discussion of packing. It allows an investigation of the physics of charge transport in organic semi­con­duc­tors (Chernyshov *et al.*, 2017 ▸; Sosorev, 2017 ▸; Ji *et al.*, 2018 ▸). The next two sections with discuss poly­mor­phism, which is the rival of crystal structure engineering.

## Polymorphism and charge transport

Polymorphism, defined as the occurrence of more than one crystal structure for a given mol­ecule, is very common among organic com­pounds (Braga *et al.*, 2010 ▸; Lecomte, 2021 ▸); organic semi­con­duc­tors are no exception. Polymorphism offers the opportunity to study the influence of mol­ecular packing in a crystal on charge transport for exactly the same organic semi­con­duc­tor (Chung & Diao, 2016 ▸; Riera-Galindo *et al.*, 2018 ▸; Gentili *et al.*, 2019 ▸). In any case, crystal structures are of the upmost importance, as illustrated for the emblematic case of rubrene, for which three crystallographic forms are known (see Table 1 ▸). Only one of the three, *i.e.* ortho­rhom­bic rubrene, gives rise to a substantial charge carrier mobility value, on the order of 20 cm^2^ V^−1^ s^−1^, illustrating once more the crucial importance of crystal structures on charge transport (da Silva Filho *et al.*, 2005 ▸; Jurchescu *et al.*, 2006 ▸; Bergantin & Moret, 2012 ▸; McGarry *et al.*, 2013 ▸; Hathwar *et al.*, 2015 ▸; Ren *et al.*, 2017*b* ▸). Inter­estingly, the crystal structures and charge-transport properties have also been determined for the isotopically substituted rubrene-*d*_28_ and the fully substituted ^13^C-rubrene (Xie *et al.*, 2013 ▸; Ren *et al.*, 2017*a* ▸). Polymorphism impacts also the properties of num­erous other semi­con­duc­tors, notably TIPS-PEN (Diao *et al.*, 2014*a* ▸) and is widely considered as a phenomenon occurring in bulk. However, the presence of a substrate surface during the crystallization process has to be taken into account, as discussed below.

## Polymorphism at surfaces and inter­faces

One peculiarity of charge transport in organic semi­con­duc­tors is that it takes place at the inter­face with a flat and impenetrable dielectric layer in the case of thin film transistors (Schweicher *et al.*, 2020 ▸). Such a rigid wall positions the centre of mass of the mol­ecules at a given distance and, consequently, mol­ecules adopt crystal motifs that differ from their bulk crystal structures (Jones *et al.*, 2016 ▸). A 2D confinement of the mol­ecular packing with the substrate surface contributes to the formation of polymorphs (Diao *et al.*, 2014*a* ▸; Giri *et al.*, 2014 ▸; Jiang & Ward, 2014 ▸; Meldrum & O’Shaughnessy, 2020 ▸; de Oliveira Martins *et al.*, 2022 ▸; Fellah *et al.*, 2022 ▸). Importantly, substrate-induced poly­mor­phism is not caused by epitaxy because it occurs on flat and unstructured substrates (Schweicher *et al.*, 2020 ▸).

Substrate-induced poly­mor­phism is not restricted to organic semi­con­duc­tors but tends to occur more for them than for other mol­ecules (*e.g.* pharmaceutical com­pounds) (Simões *et al.*, 2018 ▸; Braun *et al.*, 2019 ▸). Two reasons can be invoked to explain this observation. On the one hand, organic semi­con­duc­tors are often high aspect ratio mol­ecules forming rather elongated unit cells for which one lattice parameter is greater than the other two. Consequently, one face of the unit cell has a considerably smaller area than the others and thus a lower surface tension than the others (Drummy & Martin, 2005 ▸). The inter­facial tension with the dielectric layer is therefore considerably decreased if the unit cell stands on its smaller area face, causing a preferential orientation that constitutes a second structural constraint ruling substrate-induced poly­mor­phism. Mol­ecules which exhibit a more globular shape, resulting in unit cells for which the faces have rather com­parable areas, present less often a preferential orientation. On the other hand, organic semi­con­duc­tors, by virtue of their conjugated π-systems, are generally more rigid and more symmetrical than general mol­ecules. Another reason is that organic semi­con­duc­tor mol­ecules have less directional bonds since they are generally devoid of hydrogen bonds and strong dipoles (Sutton *et al.*, 2016 ▸). The geometrical requirement to position their mol­ecular centre at a given distance from the rigid wall (*e.g.* the substrate surface) can thus more easily relax by adopting other conformations (Aliaga-Gosalvez *et al.*, 2019 ▸; Simbrunner *et al.*, 2021 ▸). A further feature is that the mol­ecular packing is restricted by the rigid wall, which also induces poly­mor­phism within thin films (Resel *et al.*, 2018 ▸; Bedoya-Martínez *et al.*, 2017 ▸). Considerable system-to-system variations are observed, which holds generally true for crystalline mol­ecular systems. In most cases, the substrate-induced phases are observed as being metastable (Wedl *et al.*, 2012 ▸; Jones *et al.*, 2016 ▸)

**Figure 5:**
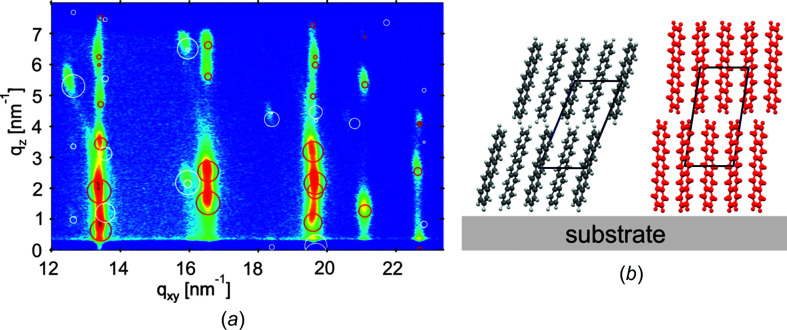
(*a*) X-ray diffraction pattern represented in a reciprocal space map of a 50 nm thick penta­cene thin film with two concomitant polymorphs. The substrate-induced (or thin film) phase and the Campbell phase are identified by red and white circles, respectively, representing the peak patterns calculated on the basis of their crystal structures. (Reproduced with permission by Springer Nature.) (*b*) Orientations of the penta­cene mol­ecules relative to the substrate surface for the Campbell phase (gray atoms) and for the substrate-induced phase (red atoms).

A quite illustrative example is given by penta­cene (Fig. 5 ▸). A new polymorph was observed within thin films prepared for transistor applications (Dimitrakopoulos *et al.*, 1996 ▸). It took more than a decade until the crystal structure of the substrate-induced phase could be solved (Nabok *et al.*, 2007 ▸; Schiefer *et al.*, 2007 ▸; Yoshida *et al.*, 2007 ▸). In the me­antime, crystal structure solution protocols have been developed for substrate-induced polymorphs, a combined experimental/theoretical approach is used based on grazing incidence X-ray diffraction (GIXD) and on mol­ecular dynamics (MD) calculations and density functional theory (DFT) (Werzer *et al.*, 2024 ▸; Lercher *et al.*, 2015 ▸; Jones *et al.*, 2017 ▸). It is also worth discussing the consequences of substrate-induced poly­mor­phism on nucleation that extends well beyond the restricted case of organic semi­con­duc­tors. It is generally admitted that homogeneous nucleation (high energy barrier) is rare and that heterogeneous nucleation (low energy barrier) is definitively more common. This is evidenced by the ratio of crystallization to melting temperature (*T*_c_/*T*_m_), which is close to unity, as routinely determined by differential scanning calorimetry (DSC) (Jackson, 1965 ▸). When substrate-induced polymorphs are present, the simple picture of the decreased energy barrier of the bulk phase due to the catalytic role of the substrate breaks down. In a first stage, heterogeneous nucleation implies the substrate-induced polymorphs and not the bulk phase. In a second stage, the latter undergoes cross-nucleation on the former. The twofold nucleation process involves two energy barriers instead of one. Two com­peting inter­actions are important at the initial state of crystallization: the mol­ecule–substrate inter­action and the mol­ecule–mol­ecule inter­action (Winkler, 2016 ▸). A strong mol­ecule–substrate inter­action can force the mol­ecules into a different orientation relative to the substrate surface, which is not the case when mol­ecule–mol­ecule inter­actions dominate (Resel, 2008 ▸). Detailed thin film growth studies have been performed on penta­cene deposited at various dielectrics (Pratontep *et al.*, 2004 ▸; Ruiz *et al.*, 2004 ▸; Luo *et al.*, 2003 ▸; Gundlach *et al.*, 1997 ▸, 1999 ▸; Dickey *et al.*, 2006 ▸; Lee *et al.*, 2007 ▸; Dinelli *et al.*, 2004 ▸; Mannsfeld *et al.*, 2009 ▸). The nucleation is associated with the formation of monolayers which are assembled by upright standing mol­ecules; subsequently, the first monolayer acts as a crystalline template for subsequent thin film growth (Pratontep *et al.*, 2004 ▸; Mannsfeld *et al.*, 2009 ▸). In the multilayer regime, the formation of islands is observed, so that layer-plus-island (Stranski–Krastanov) is present for the penta­cene thin film crystallization (Ruiz *et al.*, 2004 ▸; Luo *et al.*, 2003 ▸). Please note that considerable differences can be observed between the crystallographic order of the first mol­ecular layer at the substrate surface and the packing of the mol­ecules in the subsequent layer grown upon this first layer (Novák *et al.*, 2011 ▸; Hofer *et al.*, 2021 ▸).

For the sake of com­pleteness, one must mention crystal structure predictions by advanced com­putational methods. They constitute a formidable tool to guide the search for polymorphs, but also to rationalize the stability order of crystal forms from first principles (Woodley & Catlow, 2008 ▸; Price, 2014 ▸; Hoja *et al.*, 2019 ▸; Firaha *et al.*, 2023 ▸; Beran, 2023 ▸). Most studies have been dedicated to active pharmaceutical ingredients (APIs), although there are some reports on organic semi­con­duc­tors (Sánchez-Carrera *et al.*, 2010 ▸; Sokolov *et al.*, 2011 ▸; Obata *et al.*, 2013 ▸; Della Valle *et al.*, 2008 ▸). The field has recently been reviewed but evolves rapidly (Bhat *et al.*, 2023*a* ▸). A hierarchy of machine-learning approaches are currently used to accelerate the development of organic semi­con­duc­tors by predicting their structures and properties (Bhat *et al.*, 2023*b* ▸). However, transport properties are not only ruled by crystal structures, but also by the presence of defects and impurities that it is not possible to com­pute.

## Lattice dynamics

**Figure 6:**
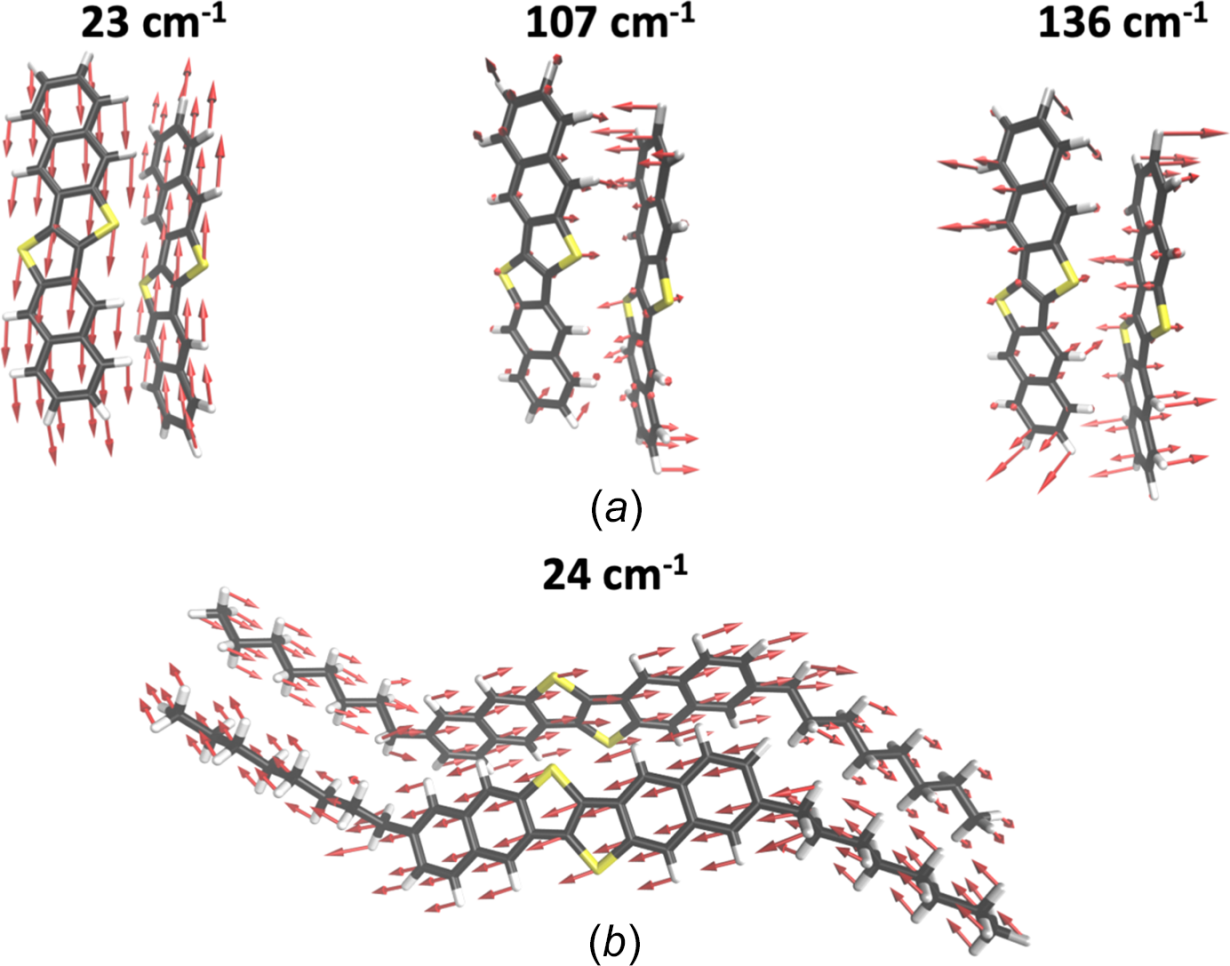
Relative displacements of neighbouring mol­ecules associated with the most detrimental phonon modes in (*a*) DNTT (23, 107 and 136 cm^−1^) and (*b*) C8-DNTT-C8 (24 cm^−1^). [Adapted from Schweicher *et al.* (2019 ▸).]

Crystals of organic semi­con­duc­tors not only have a large variety of structures but also some rich phonon dynamics that relate to structure and symmetry. Local (intra­molecular) and nonlocal (inter­molecular) phonon modes must be differentiated (Coropceanu *et al.*, 2007 ▸). Intra­molecular phonon modes, observed above 150 cm^−1^, contribute to the reorganization energy (λ) associated with electron transfer and ranges from 100 to 800 meV (Tant *et al.*, 2005 ▸). Inter­molecular phonon modes appear in the 10 to 150 cm^−1^ spectral window (Asher *et al.*, 2022 ▸; Ferrari *et al.*, 2023 ▸; Salzillo & Brillante, 2022 ▸). They cause the localization of charge carriers (so-called electron phonon couplings) over one to several mol­ecules, thus reducing drastically the charge carrier mobility values. It is only recently that thermal fluctuations have been recognized as the bottleneck limiting the performance of organic semi­con­duc­tors due to the resulting dynamic disorder (Fratini *et al.*, 2016 ▸, 2017 ▸; Giannini *et al.*, 2023 ▸; Giannini & Blumberger, 2022 ▸). But not all phonon modes are equally important. Some ‘killer’ modes, those presenting a large amplitude coupled with a large electron–phonon coupling value, occurring at low frequency, give rise to larger fluctuations of transfer integrals than others, as illustrated in Fig. 6 ▸ in the case of DNTT and C8-DNTT-C8 (Schweicher *et al.*, 2019 ▸; Stoeckel *et al.*, 2021 ▸; Banks *et al.*, 2023 ▸). Inter­molecular phonon modes are essentially probed by inelastic neutron scattering or by any low-frequency vibrational spectroscopy technique (Raman, THz). Data analysis implies knowledge of the crystal structure to fit the vibrational spectra and extract phonon modes. Recent results on a series of organic semi­con­duc­tors show that the harmonic vibration potential hypothesis breaks down and that inter­molecular phonon modes inter­act (Asher *et al.*, 2022 ▸; Benshalom *et al.*, 2023 ▸). This brief discussion of crystal dynamics illustrates that crystal structure elucidation is again an enabling step for further physical studies. Intra- and inter­molecular vibrations not only shape charge-transport physics, but also trigger phase transitions, as will be discussed in the next section.

## Crystal-to-crystal transitions

**Figure 7:**
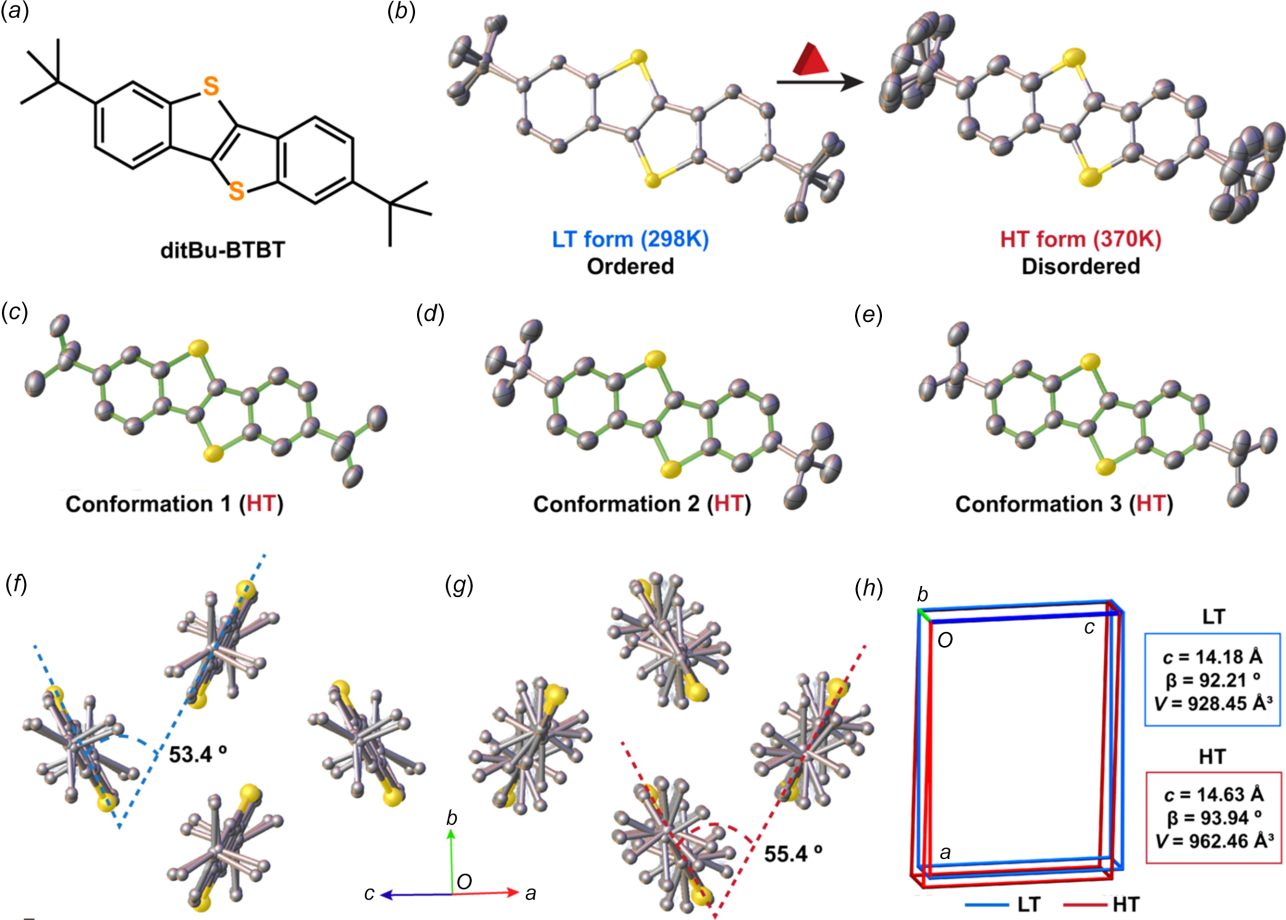
Single-crystal-to-single-crystal polymorphic transition in ditBu-BTBT from the LT to the HT form, showing (*a*) the mol­ecular structure of ditBu-BTBT, (*b*) the order-to-disorder transition of the side –ditBu groups and (*b*)/(*d*)/(*e*) the three isolated conformations of the HT disordered phase. (*f*) The change in the herringbone dihedral angle in the (*f*) LT and (*g*) HT forms. H atoms have been omitted for clarity and better presentation. (*h*) Overlay of the unit cells and significant changes of the unit-cell parameters before and after the polymorphic phase transition (Chung *et al.*, 2018*a* ▸).

Polymorphs are most often obtained from an amorphous phase, either a gas phase, a solution or a melt state. But polymorphs transform also into each other by crystal-to-crystal phase transitions, that can be either reversible or irreversible, and that are strongly kinetically hindered. In most cases, the crystal structures of two polymorphs are structurally too different to allow a direct inter­conversion without passing through an inter­mediate disordered phase (Chung *et al.*, 2019 ▸, 2020 ▸). A few exceptions exist, notably the case of ditBu-BTBT, which exhibits both a low- and a high-temperature form that inter­convert between 340 and 350 K by a concerted mech­a­nism reminiscent of Martensitic transitions. When inspecting both crystal structures, one immediately notices their structural similarity, except for the *tert*-butyl side groups that present some disorder (Fig. 7 ▸) (Chung *et al.*, 2018*a* ▸,*b* ▸; Park & Diao, 2020 ▸). Compelling Raman spectroscopic evidence corroborates that the phase transition is triggered by partial rotation of the *tert*-butyl side groups and that this specific phonon mode drives the phase (Asher *et al.*, 2022 ▸, 2023 ▸). In some cases, solvent vapour annealing (SVA) – a post deposition treatment of thin films – is used to trigger phase changes and, in most cases, transition towards the thermodynamic equilibrium state is initiated (Jones *et al.*, 2015 ▸). But solvent vapour annealing can also induce a substantial improvement of structural order in terms of thin film morphology and crystalline properties (Lee *et al.*, 2007 ▸; Dickey *et al.*, 2006 ▸). Crystal-to-crystal phase transitions are much more than laboratory curiosities because they allow the identification of the phase-transition mech­a­nisms that cause them, thanks to the availability of the crystal structures. This conclusion can be extended to the most ordered liquid crystal phases, such as the smectic E phase (Ferrari *et al.*, 2023 ▸). The dynamics of crystals is gaining an increasing importance because it contributes to the mechanical and electronic properties, probably as much as the structures (Davies *et al.*, 2023 ▸; Awad *et al.*, 2023 ▸; Das *et al.*, 2020 ▸).

## Conclusions and perspectives

This review presents a large portfolio of research activities that would not have been possible without initial crystal structure elucidation. Novel mol­ecular materials design, supra­molecular inter­action studies, crystal engineering, poly­mor­phism studies, spectroscopic analysis, lattice dynamics studies and charge-transport physics heavily rely on crystal structures, in bulk, but also at the inter­face with a substrate that acts as a rigid wall imposing geometrical constraints causing the occurrence of some substrate-induced phases. The latter requires the development of new crystal structure elucidation methods based on GIXD data obtained at synchrotron facilities. One future challenge deals with the control of nucleation and growth through better-defined crystallization conditions, implying also time-resolved structural elucidation to monitor the formation of transient phases. Structural elucidation from bulk single crystals is and will continue to play a pivotal role, but there is also a demand for the development of time- and space-resolved X-ray diffraction methods.

## Figures and Tables

**Table 1:** Crystallographic data for three polymorphic forms of rubrene (Bergantin & Moret, 2012 ▸)

	Ortho­rhom­bic rubrene	Monoclinic rubrene	Triclinic rubrene
Temperature (K)	175	173	173
Formula	C_42_H_28_	C_42_H_28_	C_42_H_28_
Formula weight	532.68	532.64	532.64
Crystal system	Ortho­rhom­bic	Monoclinic	Triclinic
Space group	*Cmca*	*P*2_1_/*c*	*P* 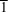
*a* (Å)	26.828 (4)	8.7397 (17)	7.0196 (14)
*b* (Å)	7.1810 (10)	10.125 (2)	8.5432 (17)
*c* (Å)	14.306 (2)	15.635 (3)	11.948 (2)
*V* (Å^3^)	2756.1 (7)	1383.3 (5)	683.5 (2)
α (°)	90	90	93.04 (3)
β (°)	90	90.98 (3)	105.58 (3)
γ (°)	90	90	96.28 (3)
*Z*	4	2	1
*D*_c_ (Mg m^−3^)	1.284	1.279	1.294
μ (mm^−1^)	0.073	0.072	0.073
2θ range (°)	6.46–52.74	5.22–54.96	4.82–54.94
Reflections collected	10119	10912	3096
Unique reflections	1434	3168	3096
GoF (obs/all)	1.057	1.099	1.196
*R*_F_^obs^ [*I* > 2σ(*I*)]	0.0403	0.0494	0.0672
*wR*_F_^all^	0.1002	0.1203	0.2149
Δρ_min_/Δρ_max_ (e Å^−3^)	0.22/−0.20	0.23/−0.18	0.25/−0.24
CCDC No.	605650	726175	726176
